# LARAMED: A Laboratory for Radioisotopes of Medical Interest

**DOI:** 10.3390/molecules24010020

**Published:** 2018-12-21

**Authors:** Juan Esposito, Diego Bettoni, Alessandra Boschi, Michele Calderolla, Sara Cisternino, Giovanni Fiorentini, Giorgio Keppel, Petra Martini, Mario Maggiore, Liliana Mou, Micòl Pasquali, Lorenzo Pranovi, Gaia Pupillo, Carlos Rossi Alvarez, Lucia Sarchiapone, Gabriele Sciacca, Hanna Skliarova, Paolo Favaron, Augusto Lombardi, Piergiorgio Antonini, Adriano Duatti

**Affiliations:** 1 Legnaro National Laboratories, Italian National Institute for Nuclear Physics (LNL-INFN), Viale dell’Università, 2, 35020 Legnaro (PD), Italy; juan.esposito@lnl.infn.it (J.E.); diego.bettoni@lnl.infn.it (D.B.); michele.calderolla@lnl.infn.it (M.C.) sara.cisternino@lnl.infn.it (S.C.); giorgio.keppel@lnl.infn.it (G.K.); mario.maggiore@lnl.infn.it (M.M.); liliana.mou@lnl.infn.it (L.M.); micol.pasquali@lnl.infn.it (M.P.); lorenzo.pranovi@lnl.infn.it (L.P.); gaia.pupillo@lnl.infn.it (G.P.); carlos.rossi.alvarez@lnl.infn.it (C.R.A.); lucia.sarchiapone@lnl.infn.it (L.S.) gabriele.sciacca@lnl.infn.it (G.S.); hanna.skliarova@lnl.infn.it (H.S.); paolo.favaron@lnl.infn.it (P.F.); augusto.lombardi@lnl.infn.it (A.L.); piergiorgio.antonini@lnl.infn.it (P.A.); dta@unife.it (A.D.); 2 Department of Physic and Earth Science, University of Ferrara, Via Saragat, 1, 44122 Ferrara, Italy; fiorentini@fe.infn.it; 3 Department of Morphology, Surgery and Experimental Medicine, University of Ferrara, Via L. Borsari, 46, 44121 Ferrara, Italy; alessandra.boschi@unife.it; 4 Department of Chemical and Pharmaceutical Sciences, University of Ferrara, Via L. Borsari, 46, 44121 Ferrara, Italy

**Keywords:** Cyclotrons, medical radioisotopes, radiopharmaceuticals, targets

## Abstract

The widespread availability of novel radioactive isotopes showing nuclear characteristics suitable for diagnostic and therapeutic applications in nuclear medicine (NM) has experienced a great development in the last years, particularly as a result of key advancements of cyclotron-based radioisotope production technologies. At Legnaro National Laboratories of the National Institute of Nuclear Physics (LNL-INFN), Italy, a 70-MeV high current cyclotron has been recently installed. This cyclotron will be dedicated not only to pursuing fundamental nuclear physics studies, but also to research related to other scientific fields with an emphasis on medical applications. LARAMED project was established a few years ago at LNL-INFN as a new research line aimed at exploiting the scientific power of nuclear physics for developing innovative applications to medicine. The goal of this program is to elect LNL as a worldwide recognized hub for the development of production methods of novel medical radionuclides, still unavailable for the scientific and clinical community. Although the research facility is yet to become fully operative, the LARAMED team has already started working on the cyclotron production of conventional medical radionuclides, such as Tc-99m, and on emerging radionuclides of high potential medical interest, such as Cu-67, Sc-47, and Mn-52.

## 1. Introduction

Radiopharmaceuticals (RPs) are molecular tools that nuclear medicine (NM) routinely employs for the diagnosis and treatment of diseases. The success of NM is deeply rooted in the close collaboration between nuclear physics, chemistry, biology, and pharmacology. This multidisciplinary effort guarantees to efficiently combine the biological and pharmacokinetic properties of molecules with the nuclear properties of radioisotopes. This combination makes it possible to visualize the RP journey inside the living organism and to monitor its biomolecular interactions with subcellular structures, thus disclosing diagnostic information at a very fundamental level. When the nuclear characteristics of a radionuclide allow simultaneous performance of both diagnosis and therapy, theranostic applications are also another potential option.

Progress in NM is always tightly linked to the continuous development of nuclear processes for the efficient production of novel radionuclides. As rule of thumb, only through the constant supply of novel radionuclides can NM progress and find innovative solutions to still open clinical questions. In this context, the Laboratory of Radioisotopes for Medicine (LARAMED) project started at the Legnaro National Laboratories (LNL) near Padua, Italy, with the aim to establish an advanced science and technology hub to study and develop new or more efficient methods for the production of radionuclides with high potential medical interest.

LNL is one of the four Italian laboratories belonging to the National Institute of Nuclear Physics (INFN) and the general research effort of the LARAMED project is entirely focused on the extensive utilization of the high performance 70p cyclotron (70 MeV, 750 μA max), which was recently commissioned within the broader framework of the so-called SPES (Selective Production of Exotic Species) project [1,2]. It is worthy to note that the primary focus of the SPES project is the fundamental research activities in nuclear physics and astrophysics with exotic ion beams alongside with applied physics to medicine, including the production of unprecedented medical radionuclides. Since only a few examples of this type of high-energy, high-current cyclotrons are currently operating around the world, it becomes immediately apparent the important role that LARAMED could play in the global scenario related to the production of radionuclides of medical interest.

LARAMED research activity is envisaged to cover different topics, ranging from basic nuclear physics (experimental measurements of excitation functions), to target technology (design and manufacturing of high-power targets for proton irradiation) and radiochemistry (development of highly-automated separation/purification techniques and labelling studies of new biological carriers). The synergy between these skills resulted in high-level research on the cyclotron production of conventional and emerging radionuclides, as summarized in this letter, introducing a brief overview of the LARAMED research programs currently underway and a description of the new facility.

## 2. The LARAMED Facility

**Figure 1:**
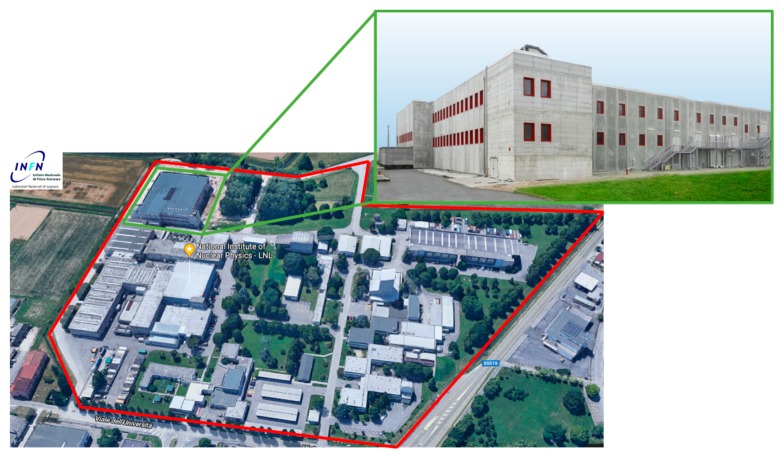
Aerial photo of the INFN-LNL area (**left**) and a zoom on the SPES building (**right**).

**Figure 2:**
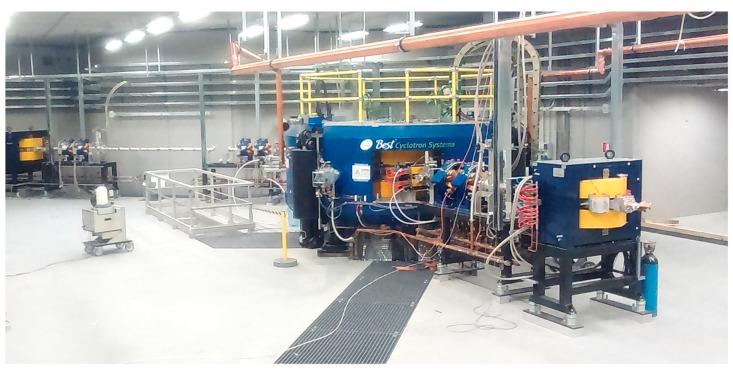
The new high-energy (70 MeV) and high-current (750 µA) proton-beam cyclotron installed at LNL-INFN (Legnaro). One of the two main switching magnets may be seen on the right.

**Figure 3:**
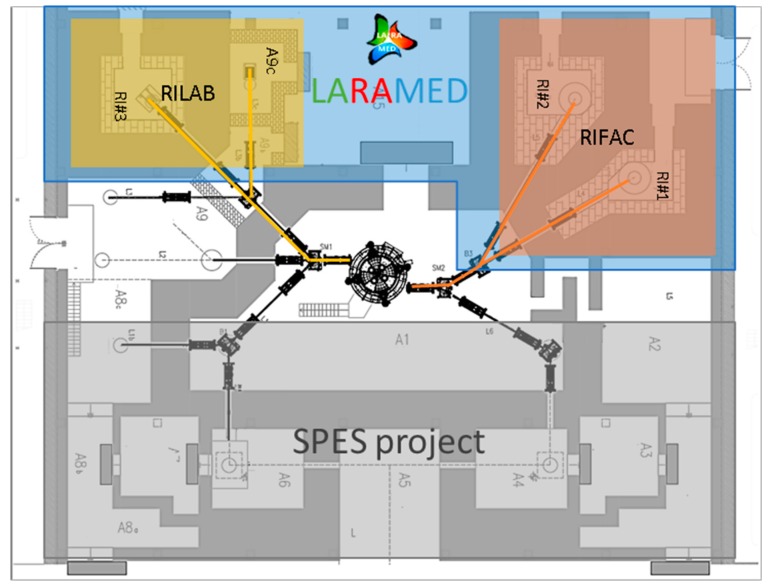
The layout of the SPES building (underground level) showing the cyclotron with outward beam-lines. In evidence, the LARAMED section (colored in blue) comprising four bunkers (RI#1, RI#2, RI#3, A9c). This area is divided in two separate sections: The Radioisotopes Laboratory (RILAB) and the Radioisotope Factory laboratory (RIFAC). On the other side, the SPES bunkers (colored in gray) are dedicated to the production of neutron-rich unstable nuclei for research in fundamental nuclear physics and astro-physics, as well as for radioisotopes production using the Isotope Separator On-Line (ISOL) technique.

The core of the SPES and LARAMED projects is the model BCSI 70p cyclotron, manufactured by Best^TM^ Cyclotron System Inc. (Vancouver, BC, Canada), installed in the central bunker located at the underground floor of the SPES building (Figure 1 and Figure 2). The cyclotron is able to deliver simultaneously two beams of accelerated protons, with a tunable energy in the range 35 MeV to 70 MeV, and a maximum accelerated current of 750 µA, with a double extraction system (Figure 2). Each extraction beamline is further split by two main switching magnets in additional sub-lines, which will be channelled towards other irradiation bunkers dedicated to different research purposes (Figure 3, Table 1). Two beamlines, assigned to LARAMED experimental activities at the so-called RILAB (RadioIsotopes LABoratory) facility at the underground level (Figure 3), are currently under construction, including new target stations specifically dedicated to the irradiation of solid targets. At the opposite side, pointing towards the RIFAC (RadiIoisotopes FACtory) facility, two additional beamlines will be used for the production of massive amounts of radioisotopes to be distributed to hospitals and clinical departments for both routine and clinical research purposes. It is anticipated that this activity will be pursued in conjunction with some private partnership.

The main features (beam energy and current) planned for each of the four proton beamlines aimed at the radioisotope research and production activities at the LARAMED facility are listed in Table 1.

**Figure 4:**
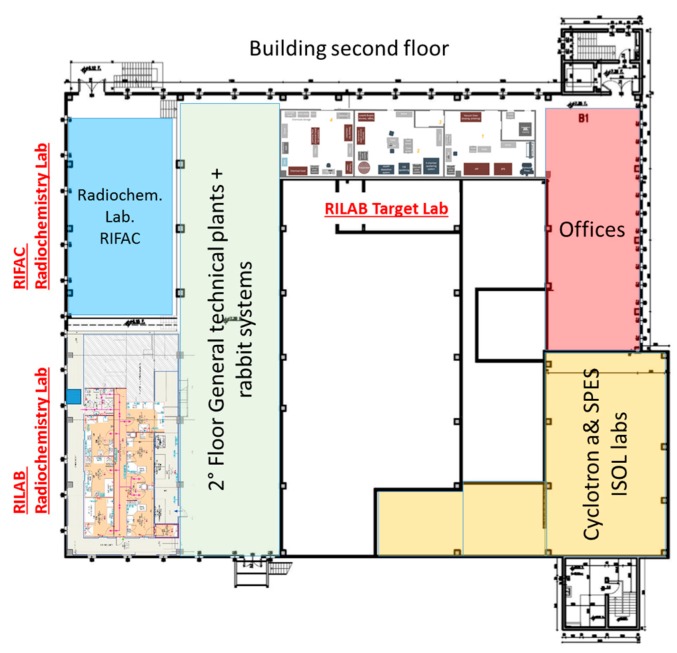
The layout of the LARAMED laboratories at the second floor of the SPES building.

Figure 4 reports a schematic summary layout of the LARAMED laboratories’ set up at the upper floor above the cyclotron vault. This area comprises:

- RILAB radiochemistry labs, designed for R&D (Research and Development) activities on radioisotope production, isolation, purification, and quality control;
- RILAB-Target lab, designed for developing and manufacturing innovative solid target systems for both nuclear physics and medical radioisotopes production; and
- RIFAC labs, for radioisotope/radiopharmaceutical production.

## 3. Ongoing LARAMED Research Programs

The LARAMED project is foreseen to shape up as a multidisciplinary research program requiring different competencies, such as nuclear physics, spectroscopy, material science, engineering, chemistry, and radiochemistry. It is currently pursued according to the following research lines: (1) Determination and re-evaluation of nuclear cross-sections for the production of novel or already established radioisotopes of relevant interest for NM; (2) design and manufacturing of targets suitable for use under high-power proton beams; (3) development of fully-automated radiochemical procedures for target processing, separation, and purification of medical radioisotopes; (4) design and development of novel radiopharmaceuticals for targeted imaging and therapy in oncology; and (5) the study of procedures for the efficient recovery of costly enriched target materials.

Although the irradiation and laboratory facilities are still not fully operational, a number of investigational studies have been carried out, or are still underway, at LNL, under the framework of the LARAMED program. In particular, the two related projects, APOTEMA (Accelerator-based Production of Technetium/-Molybdenum for medical Applications) and TECHN-OSP (TECHNetium direct-production in hOSPital), dealt with the production of the NM workhorse radionuclide, ^99m^Tc, by cyclotrons. The cyclotron production of emerging radionuclides, such as ^67^Cu, ^47^Sc, and ^52^Mn, have been investigated within the COME (COpper MEasurement), PASTA (Production with Accelerator of Sc-47 for Theranostic Applications), and METRICS (Multimodal pET/mRi Imaging with Cyclotron-produced ^52/51^Mn iSotopes) projects, respectively. Moreover, technological R&D focused on innovative targets for medical radionuclides production is investigated with the E-PLATE (Electrostatic Powder pLating for Accelerator TargEts) and TERABIO (innovative technologies for radionuclide ThERApy and BIOimaging) projects (Table 2). The successful implementation of the above-mentioned projects would not have been possible without the invaluable collaboration of the following institutions: The Department of Nuclear Medicine of Sant’Orsola-Malpighi Hospital, Bologna, Italy and of Sacro Cuore Don Calabria Hospital, Negrar, Verona, Italy, the international institute JRC-Ispra (Joint Research Centre), Varese, Italy, and the ARRONAX centre, Nantes, France.

### 3.1. APOTEMA and TECHN-OSP: ^99m^Tc Cyclotron Production

It is worth mentioning the activities about the production of ^99m^Tc (T_1/2_ = 6.06 h, E_γ_ = 140 keV (89%)), nowadays still the most used radionuclide worldwide for conventional nuclear medicine scans, such as single photon emission computed tomography (SPECT), carried out in the framework of the INFN-funded (National Scientific Committees 5-CSN5, 2012–2017) research program, APOTEMA/TECHN-OSP.

^99m^Tc is commonly obtained from the decay of its parent nuclide, ^99^Mo (half-life 66 h), by eluting compact and transportable ^99^Mo/^99m^Tc generator systems that make it almost always available directly in nuclear medicine departments. The majority of ^99^Mo is produced in a few ageing nuclear reactors around the world, using highly enriched uranium (HEU) targets. The ^99^Mo shortage that occurred in 2009 has prompted the research community interest towards the production of ^99m^Tc by alternative ways, especially by using particle accelerators [4,5,6,7,8,9,10,11,12]. Even more recently (November 2018), the worldwide ^99^Mo/^99m^Tc generators market went through another production shortage event of production shortage due to the contemporary shutdown of NTP (Nuclear Technology Products, South Africa) facility and OPAL (Open Pool Australian Lightwater, New South Wales, Australia) reactor while the HFR (High Flux Reactor, Petten, the Netherlands) reactor was returning to service after an unexpected shutdown [13]. Moreover, the announcement about the NRU reactor (National Research Universal, Chalk River, ON, Canada) permanent shutdown has become effective since March 2018 after 60 years of service covering over 40% of the Mo-99 global market [14,15,16,17]. Within the framework of a Coordinated Research Project (CRP No. F22062) promoted by the International Atomic Energy Agency (IAEA), APOTEMA experiments, performed by INFN in collaboration with other Italian Universities and Research Institutions, were focused on the following items:

1. Evaluation of the nuclear characteristics of the most suitable nuclear reactions that could be employed for the production of ^99^Tc by cyclotrons, in particular the ^100^Mo(p,2n)^99m^Tc reaction [18,19,20,21];
2. radiochemical separation/purification processes’ optimization for extracting ^99m^Tc from the irradiated target [22];
3. assessment and impact determination of the ^99g^Tc/^99m^Tc isomeric ratios as well as other Tc-contaminants, expected in the cyclotron-produced ^99m^Tc, on the radiolabeling efficiency and imaging quality [23,24]; and
4. radiopharmaceuticals and preclinical studies [23,25,26].

Outcomes from those experiments has triggered a research interest on ^99m^Tc-radiopharmaceuticals involving part of the group in the development of new tracers and the evaluation of ^99m^Tc routine practice [27,28,29,30].

The main results were the determination of the optimal irradiation parameters in order to get an as high as possible cyclotron-^99m^Tc production by minimizing the co-production of isotopic contaminants; the design and setup of a first radiochemical module prototype, based on the solvent extraction technique, was realized accordingly to achieve the appropriate purification in high yield for the ^99m^Tc, proving the feasibility of the process and the quality of the product by performing in vivo imaging tests.

**Figure 5:**
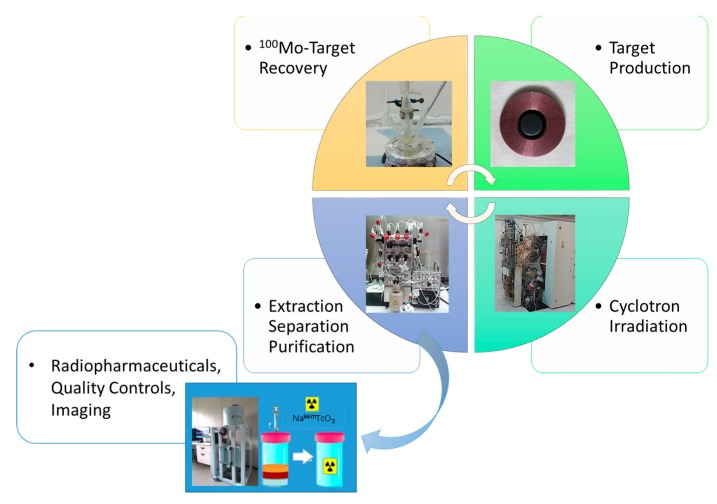
Schematic representation of the tasks accomplished within the APOTEMA and TECHN-OSP projects.

As a follow up of the APOTEMA program, the TECHN-OSP project was aimed at the technology refinement and upgrade, in order to scale up the ^99m^Tc cyclotron production, with enough to cover the needs of a medium Italian nuclear medicine department in the view of a real clinical application (Figure 5).

This required setting up a complex process involving (a) the production of high-performance highly-enriched ^100^Mo targets, (b) an upgrade of the separation and purification automatic module, and (c) development of a procedure and technology for the recovery of the irradiated target. Results from this project demonstrated that the cyclotron production of ^99m^Tc is feasible and that the quality of the resulting radionuclide is basically in compliance with the limits recently issued by the European Pharmacopeia (Ph.Eur.) monograph on cyclotron produced ^99m^Tc [24,31,32].

**Figure 6:**
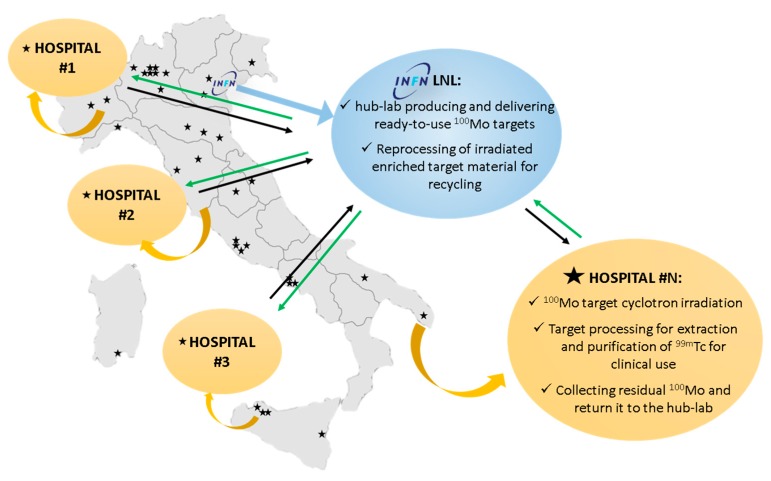
Schematic picture of the proposed “hub and spoke” approach for in-hospital cyclotron-^99m^Tc production. The green arrows represent the distribution of ready-to-use ^100^Mo targets produced by the hub-lab; the black arrows represent the used enriched target material returned from the hospitals to the hub-lab.

Finally, considering the Italian reality where about 40 hospital cyclotrons are spread over the country and are used for ^18^F daily production for Positron Emission Tomography (PET) scans, the TECHN-OSP group estimated a production cost of about 2$/mCi of the in-hospital produced ^99m^Tc [32], assuming the possibility to use the available cyclotron, with appropriate characteristics, also to produce ^99m^Tc applying a “hub and spoke” approach (Figure 6):

1. A hub-lab can produce and deliver stable ^100^Mo targets;
2. hospitals produce and separate ^99m^Tc using their cyclotrons and the automatic module we developed; and
3. hospitals send back to the hub-lab the used enriched target material for reprocessing.

The main results achieved within the APOTEMA and TECHN-OSP projects are summarized in Table 3.

### 3.2. COME: COpper Measurement

The production of ^67^Cu in, as much as possible, its radionuclide pure form has been a top priority since the early stage of the LARAMED project. The great interest in ^67^Cu comes from the very favorable nuclear properties for both diagnosis and therapy and its long half-life (T_1/2_ = 61.9 h), suitable to follow the slow biodistribution of large molecules, such as monoclonal antibodies. ^67^Cu, thanks to the combined emissions of β-particles (E_β−mean_ = 141 keV (100%)) and γ (E_γ_ = 184.6 keV (48.7%)), can be conveniently exploited for obtaining diagnostic images of internal organs and, based on this information, for tailoring a personalized therapeutic approach. For this reason, ^67^Cu is an almost ideal example of a theranostic radionuclide that can be employed simultaneously both for diagnosis and therapy. Moreover, ^67^Cu can be paired with the positron emitter, ^64^Cu (T_1/2_ = 12.701 h, E_β+mean_ = 278 keV (17.60%)), to perform low-dose PET (positron emission tomography) dosimetric studies prior to therapy. Another outstanding feature of ^67^Cu arises from the recent observation that, when administered as simple Cu^2+^ ions, it is selectively taken up by cancerous cells that, in turn, can be visualized and subsequently destroyed by a cytotoxic radiation dose. These results emphasize the enormous potential of ^67^Cu for NM [33].

Currently, a reliable supply with sufficient clinical amounts of ^67^Cu throughout the world does not exist. The production of ^67^Cu still presents considerable challenges because of some unfavorable parameters characterizing the nuclear reactions that have been investigated. In particular, the most intensively studied reaction has been the nuclear process, ^68^Zn(p,2p)^67^Cu. Using this route, at P. Scherrer Institute (PSI) in Switzerland and at Brookhaven National Laboratories in the United States, small amounts of ^67^Cu are occasionally produced [34]. Millicuries amounts of ^67^Cu are regularly produced only at the National Isotope Development Center of Oak Ridge National Laboratory based on the ^68^Zn(p,2p)^67^Cu nuclear reaction [35], carried out with the use of high-energy proton accelerators (200 MeV), and at the Idaho Accelerator Center [36], based on the photonuclear reaction, ^68^Zn(γ,p)^67^Cu by e-linac.

**Figure 7:**
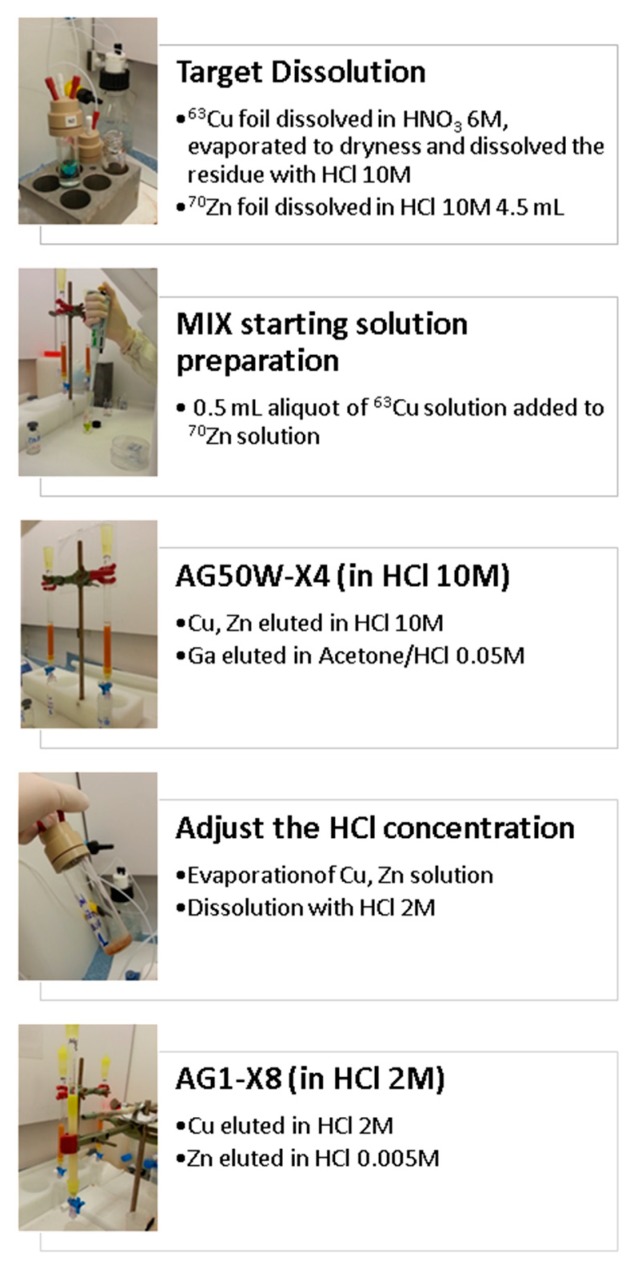
Schematic description with photographs of the key steps of the radiochemical separation process developed for the COME project.

In this regard, the aim of the COME project, funded by INFN (CSN3, 2016) and participating in a Coordinated Research Project promoted by the IAEA (CRP No. F22053), was to experimentally determine the cross-section of the ^70^Zn(p,x)^67^Cu reaction in the still unexplored proton energy region above 35 MeV [37]. An added value of this project was the development of a high-yield chemical separation procedure of Cu from Zn and Ga elements, optimized for the cross-section measurement (Figure 7). The COME project successfully identified the optimal experimental conditions to maximize the ^67^Cu yield that can be efficiently employed to produce ^67^Cu with the 70 MeV cyclotron installed at LNL (results under publication elsewhere). Results of the studies carried out in the COME project brought to the idea of a special target design that allows the maximization of ^67^Cu production while minimizing the ^64^Cu contamination, submitted as a patent application (P2974IT00) [38].

### 3.3. PASTA: Production with Accelerator of Sc-47 for Theranostics Applications

Scandium-47, as ^67^Cu, is another example of a theranostic radionuclide, having nuclear properties that are intrinsically suitable for both therapy and diagnosis since it simultaneously decays with β-and γ-emissions (Table 2). As already mentioned, the potential advantage of ^47^Sc and of theranostic radionuclides in general, lies in the possibility to applicate the so-called “personalized medicine” approach. This last factor allows the selection of patients that respond to a specific therapeutic treatment, by performing low-dose imaging studies with the same radiopharmaceutical *a priori*.

**Figure 8:**
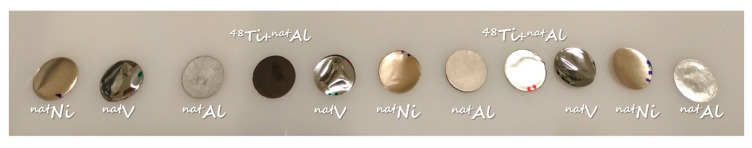
Photograph of a typical stacked-foils target of the PASTA project.

The production of ^47^Sc in sufficient quantities and at a reasonable cost is challenging. The PASTA project, funded by INFN (CSN5, 2017–2018), is thus focused on the investigation of a suitable nuclear process to obtain ^47^Sc in adequate amounts for medical use. The most promising proton-induced nuclear reactions cross-section, such as ^50^Ti(p,α), ^49^Ti(p,2pn), ^48^Ti(p,2p), and ^nat^V(p,x), are under measurements by using stacked-foils targets composed by a set of thin metal foils (Figure 8). The individuation of the optimal irradiation parameters to minimize the production of contaminant radionuclides will be based on the experimental results, actually under analysis. Moreover, a radiochemical separation of Sc from Ti based on solid phase extraction chromatography has been also implemented within this project.

### 3.4. METRICS: Multimodal pET/mRi Imaging with Cyclotron-Produced ^52/51^Mn iSotopes

Multimodality imaging is a diagnostic technique that combines morphological and functional information for improving the power of current imaging methods [39]. PET or SPECT with CT (computed tomography) are currently the most popular multimodal imaging technologies in this field. Moreover, the combination of PET or SPECT with MRI (magnetic resonance imaging) is a newly-established technology. However, this multimodal technology usually involves the use of two different compounds, a contrast agent for MRI (i.e., Gadolinium) and a radioactive tracer for PET/SPECT, generating a mismatch in the diagnostic information content. In this regard, we want to investigate the possibility and the effect of having a molecular fusion between PET and MRI by using a bimodal probe, a molecule able to be detected by both techniques. The same compound will act as contrast and radioactive agents, preserving both the paramagnetic and nuclear characteristics. Manganese is the only transition element having paramagnetic properties suitable for MRI and two manganese isotopes, ^52^Mn and ^51^Mn, positron emitters that could be employed as PET tracers (Table 2). The scope of the METRICS project, funded by INFN (CSN5, 2018–2020), is to develop a perfect molecular matching between PET and MRI using paramagnetic and radioactive manganese isotopes to afford an unprecedented type of PET/MRI hybrid imaging and to develop the technology, target, and separation module to self-produce by cyclotron the ^52^Mn. So far, natural chromium targets have been successfully produced and tested under a proton beam up to 50 µA (~ 1 kW) for thermomechanical tests, while manganese paramagnetic complexes have been synthesized and characterized.

### 3.5. E-PLATE: Electrostatic Powder pLating for Accelerator TargEts

**Figure 9:**
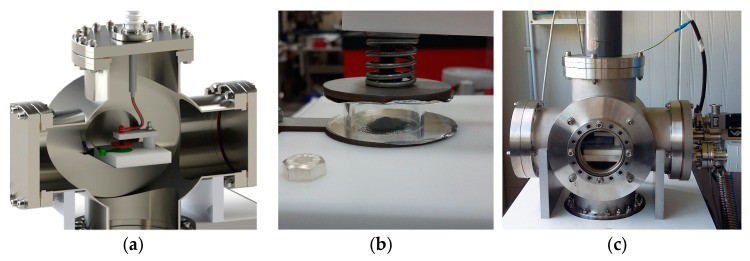
HIVIPP deposition system: (**a**) 3D general scheme; (**b**) photo of the deposition chamber: Substrates attached to the two high voltage electrodes in parallel and powders contained by the quartz cylinder in between; (**c**) photo of the working system.

The target preparation is often crucial for the success of both nuclear physics experiments and medical radionuclide production. When enriched isotopes are used for target preparation, the technique providing minimal material losses is required. For refractory metals, like Ti, Mo, W, Zr, and Hf, standard target preparation techniques are often inefficient. The HIVIPP (HIgh energy VIbrational Powder Plating) method [40] provides a solution to both described problems: It allows the deposition of even “problematic” refractory metals with minimal losses of the starting material. In Figure 9, a 3D representation of the HIVIPP deposition system and pictures of the working system, installed at LNL-INFN in the framework of the E-PLATE project, are presented.

The main goal of the E-PLATE project, funded by INFN (CSN5, 2018–2019), is to identify the reason of the thickness limitation (“saturation” effect) of the HIVIPP deposit, and overcome it, in order to allow the preparation of the targets, such as for nuclear cross-section measurements and medical radionuclides production. An extensive study of the parameters influencing the deposition, like the powders’ size and oxidation level, electric field, substrate properties, etc., is proposed in order to have the opportunity to adapt the process for different types of metallic powder. Meanwhile, after the preliminary HIVIPP deposition tests with Ti-nat powders, more than 20 ^48^Ti enriched titanium targets of a 0.5–4 µm thickness were produced and utilized for cross-section measurements in the framework of the PASTA project.

### 3.6. TERABIO: innovative technologies for radionuclide ThERApy and BIOimaging

A follow-up of the LARAMED project, called TERABIO, was further submitted and granted as a special project in 2016 by the Italian Ministry of Research (MIUR). The focus of the TERABIO project is on the technological R&D on high power targets, allowing the production of medical radionuclides in adequate quantities.

In order to maximize the nuclear reaction yield, the production should be performed at maximum proton currents. This means that the target system should provide high efficiency of the heat dissipation. Moreover, it should be always kept in mind that the target backing material should be chemically inert in respect to the target dissolution solution, otherwise a dedicated impurities separation process (usually consuming time after irradiation) should be developed.

The LARAMED group, for the case of ^99m^Tc production, has developed a novel target preparation approach consisting of Mo deposited by magnetron sputtering onto chemically inert sapphire or synthetic diamond, brazed to a high thermal conductivity metallic holder. The targets produced by this way have shown excellent thermomechanical stability under the beam with the heat power density up to ~1 kW/cm^2^ [41]. This target preparation method has been patented by INFN (patent number P1183PC00) [42]. The magnetron sputtering approach has been successfully employed also for the ^nat^Y target preparation for production of ^89^Zr (>0.6 kW/cm^2^).

In addition to this, a particular press-sintering technique called spark plasma sintering (SPS) was successfully used for the preparation of cyclotron solid targets of ^100^Mo, ^nat^Cr, and ^nat^Y for the production of ^99m^Tc, ^52^Mn, and ^89^Zr correspondingly. The SPS technique allowed simultaneous sintering and press-bonding to a metallic backing plate with low losses of the material during processing. Such Mo targets on copper and copper covered by protective layer backing have shown high-performance under proton irradiation (>1.3 kW/cm^2^) without any evidence of thermomechanical damage.

The design and construction of a high power solid target (more than 10 kW) is a key critical technological problem. Our approach is a synergy of ANSYS thermomechanical modeling (ANSYS, Inc. Canonsburg, PA, USA) with experimental study of target prototypes (or parts of the target) prepared by proposed methods, like target material deposition, vacuum brazing for backing, deposition of protective layers to avoid oxidation, etc.

## 4. Conclusions

The LARAMED project has as major objective the establishment at LNL of a research and production facility of conventional and emerging relevant medical radionuclides to be distributed to hospitals and clinical departments, both for clinical research purposes and routine use in patients’ treatment. Outcomes from ongoing activities may allow the development of a whole procedure for the production of ^67^Cu, as soon as the necessary infrastructures and equipment, currently under installation, will be finally completed. These expected remarkable results strongly point out that LNL might become the first European supplier of this outstanding radionuclide, promoting the essential research for the development of innovative cancer therapies. In particular, the development of a target technology suitable for operating with high-current, high-energy proton beams is required for the production of sufficient amounts of ^67^Cu for further medical studies. The results obtained in previous R&D on targetry will be combined with novel ideas related to exotic cooling configurations, like diamond-based backing, metal-diamond composite backing, and different cooling channel configuration (comprising 3D-printed prototypes). Although LARAMED will be a public research facility, joint ventures with private companies interested in the radioisotopes cyclotron production programs is foreseen. Such a new view may indeed represent a first step evolution of the LNL activities, traditionally devoted to basic nuclear physics and applied studies, towards a new synergy, where society will be the final beneficiary. At last, it must also be outlined that such a new research framework at LNL may also lead to the training of young researchers engaged in a multidisciplinary/technological endeavor towards new frontiers that, we think, is a project-added value.

## Figures and Tables

**Table 1:** Proton-beam characteristics of the four LARAMED beamlines with a tunable energy of 35–70 MeV each.

Facility	Target Station/Bunkers	Expected Current
RIFAC	RI#1 RI#2	500 µA 500 µA
RILAB	RI#3 A9c	300 µA 100 nA

**Table 2:** Nuclear data of interest associated to the investigated radionuclides (RN) [3].

Project	RN	Half-Life	E_γ_ (keV)	I_γ_ (%)	E_β−mean_ (keV)	I β− (%)	E_β+mean_ (keV)	I β+ (%)
APOTEMA/TECHN-OSP	^99m^Tc	6.0072 h (9)	140.511 (1)	89 (4)				
COME	^67^Cu	61.83 h (12)	184.577 (10)	48.7 (3)	141 (13)	100 (6)		
PASTA	^47^Sc	3.3492 days (6)	159.381 (15)	68.3 (4)	162.0 (21)	100 (8)		
METRICS	^52^Mn	5.591 days (3)					242 (5)	29.4 (4)
	^51^Mn	46.2 m (1)					962.8 (5)	97.09 (3)

**Table 3:** Summary of the main results achieved within the APOTEMA and TECHN-OSP projects [18,19,20,21,22,23,24,25,26,32]. * SPS = Spark Plasma Sintering; ^§^ MEK SE = Solvent Extraction with Methyl Ethyl Ketone.

Tasks	Main Results
Identification of the optimal irradiation parameters	- Proton beam energies: 15–20 MeV; - Irradiation time: 3–6 h; - Mo-100 enrichment level > 99%.
Target configuration	- Thick (300 µm) Mo-100 (99.86%) metal target produced by SPS * on a Cu/Au backing; - Thermomechanical stability up to 1.3 kW.
Target processing	- Dissolution + MEK SE ^§^ in an automatic module; - Processing time 60 min; - Recovery yield 93%.
Mo-100 target recovery in metallic form	- Decomposition of Na_2_MoO_4_ to MoO_3_; - MoO_3_ reduction to Mo metal (two-steps hydrogen reduction in a dedicated static hydrogen gas overpressure reactor); - Total overall recovery yield > 85%.
